# Exploring Formation and Control of Hazards in Thermal Processing for Food Safety

**DOI:** 10.3390/foods14132168

**Published:** 2025-06-21

**Authors:** Zeyan Liu, Shujie Gao, Zhecong Yuan, Renqing Yang, Xinai Zhang, Hany S. El-Mesery, Xiaoli Dai, Wenjie Lu, Rongjin Xu

**Affiliations:** 1 School of Food and Biological Engineering, Jiangsu University, Zhenjiang 212013, China; 2212318015@stmail.ujs.edu.cn (Z.L.); 2212418006@stmail.ujs.edu.cn (S.G.); 2212418018@stmail.ujs.edu.cn (Z.Y.); 2212318006@stmail.ujs.edu.cn (R.Y.); 2 School of Energy and Power Engineering, Jiangsu University, Zhenjiang 212013, China; elmesiry@ujs.edu.cn (H.S.E.-M.); dai_xiaoli1980@126.com (X.D.); lwj@ujs.edu.cn (W.L.); xurongjin@ujs.edu.cn (R.X.)

**Keywords:** thermal processing, food processing contaminants, antioxidant addition, formation, control measures

## Abstract

Thermal-processed foods like baked, smoked, and fried products are popular for their unique aroma, taste, and color. However, thermal processing can generate various contaminants via Maillard reaction, lipid oxidation, and thermal degradation, negatively impacting human health. This review summarizes the formation pathways, influencing factors, and tracing approaches of potential hazards in thermally processed foods, such as polycyclic aromatic hydrocarbons (PAHs), heterocyclic aromatic amines (HAAs), furan, acrylamide (AA), trans fatty acids (TFAs), advanced glycation end-products (AGEs), sterol oxide. The formation pathways are explored through understanding high free radical activity and multiple active intermediates. Control patterns are uncovered by adjusting processing conditions and food composition and adding antioxidants, aiming to inhibit hazards and enhance the safety of thermal-processed foods.

## 1. Introduction

Thermal treatment is commonly used to enhance the nutritional value, taste, and shelf life of food products [1,2]. However, thermal treatment can also lead to adverse consequences, including the generation of heat-derived toxic substances that do not naturally exist in food [3,4,5,6]. These substances are known as food processing contaminants, such as polycyclic aromatic hydrocarbons (PAHs) like benzo[a]pyrene, heterocyclic aromatic amines (HAAs), furan, Acrylamide (AA), trans fatty acids (TFAs), advanced glycation end-products (AGEs), and sterol oxides [7,8,9,10]. These contaminants often form through Maillard reactions, lipid oxidation, and thermal degradation, driven by high temperatures and cooking methods [11,12,13].

**Scheme 1:**
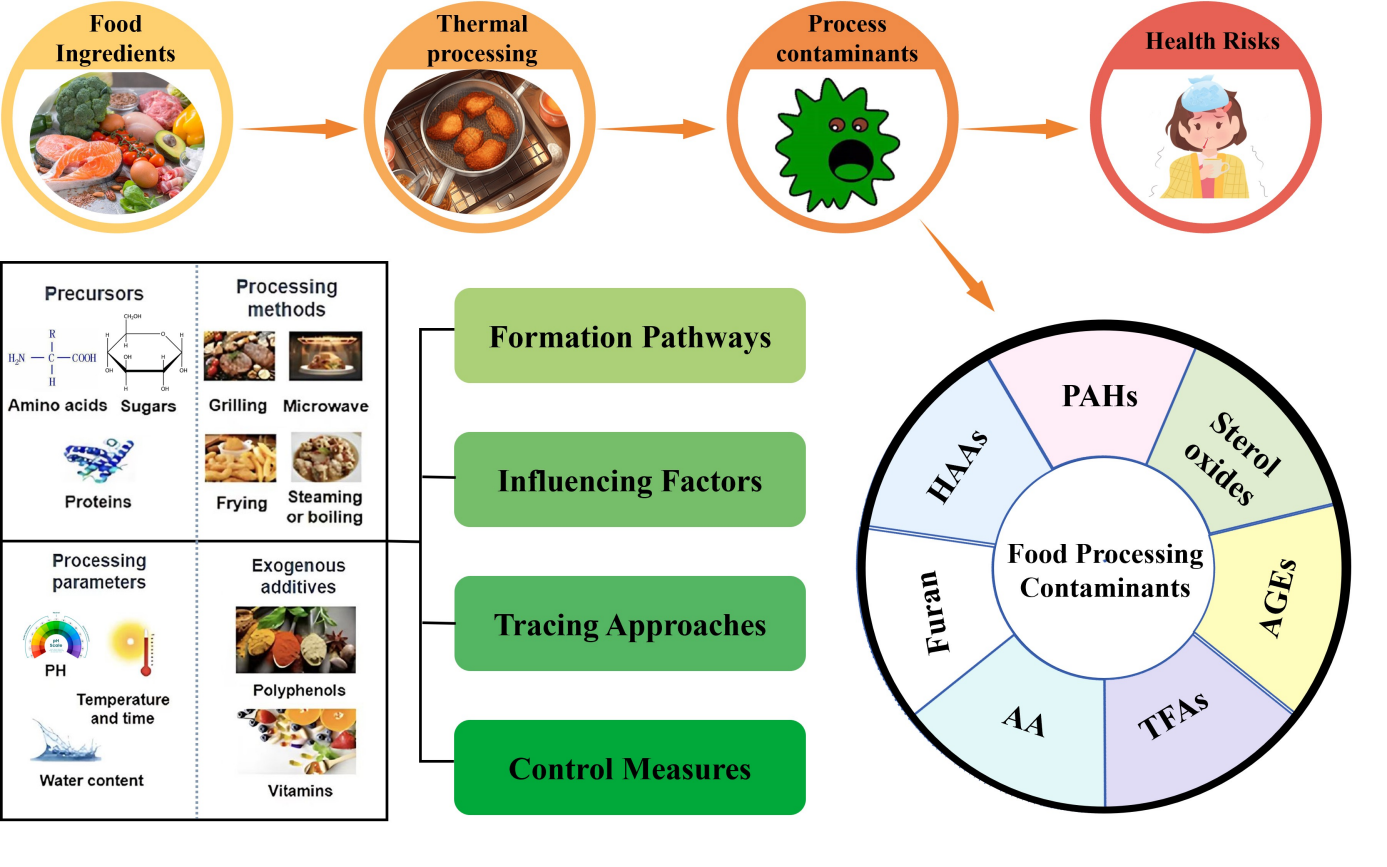
Schematic diagram of potential contaminants generated during thermal processing of food.

Long-term exposure to hazards from thermally processed foods can cause public health issues, including carcinogenicity, genotoxicity, neurotoxicity, and cardiovascular complications [14,15,16]. The International Agency for Research on Cancer (IARC) has indicated that consuming 50 g of processed meat per day can increase the incidence of pancreatic, colon, breast, and prostate cancer by 19%, 18%, 9%, and 4%, respectively. The IARC categorizes compounds based on their carcinogenicity into four groups: Group 1 (carcinogenic to humans), Group 2A (probably carcinogenic to humans), Group 2B (possibly carcinogenic to humans), and Group 3 (not classifiable as carcinogenic to humans). For instance, PAHs encompass compounds from all IARC groups: Group 1 includes benzo[a]pyrene (BaP), Group 2A includes dibenzo[a,h]anthracene, Group 2B includes chrysene, and Group 3 includes pyrene. Similarly, HAAs contain compounds classified as Group 2A and Group 2B, AA as a probable carcinogen (Class 2A), a designation reaffirmed by a World Health Organization (WHO) panel in 2002 [17,18]. In addition, epidemiological studies have shown that HAAs are involved in the occurrence of colorectal, pancreatic, bladder, and kidney cancers. Chang et al. showed that in some upper-middle-income countries, ultra-processed foods account for an average of more than 50% of people’s total energy intake. These foods contain “hydrogenated” or “partially hydrogenated” vegetable oils (trans fats). Women with high levels of trans fats in their foods nearly double their risk of breast cancer [19,20]. Therefore, this highlights the need for a deeper understanding of their formation mechanisms, health impacts, tracking methods, and risk mitigation strategies. Current efforts focus on regulatory measures, industrial practices, and consumer education, with authorities setting contaminant standards and manufacturers implementing good manufacturing practices, hazard analysis, and critical control point systems [21,22,23,24]. However, completely avoiding process contaminants remains a challenge [25,26,27,28]. This review aims to propose a comprehensive approach to address processing contaminants in thermally processed foods, identify key factors in their formation, and implement targeted interventions to minimize their presence. It provides a holistic approach covering formation mechanisms, influencing factors, tracking pathways, and control measures of food processing contaminants (PAHs, HAAs, furan, AA, TFAs, AGEs, and sterol oxides) (Scheme 1), offering integrated resources for policymakers, manufacturers, and consumers. The review seeks to contribute to ongoing discussions and drive quality enhancement in the food industry.

## 2. Polycyclic Aromatic Hydrocarbons (PAHs)

PAHs are organic compounds composed of carbon and hydrogen atoms, featuring two or more fused aromatic rings in various configurations. They are categorized by molecular weight into light PAHs (up to four rings) and heavy PAHs (more than four rings). Light PAHs are characterized by their volatility and relatively lower toxicity compared to heavy PAHs, which are more lipophilic [29,30]. The European Food Safety Authority (EFSA) has identified four major PAHs—BaP, benzo[a]anthracene (BaA), benzo[b]fluoranthene (BbF), and chrysene (Chry)—as markers for the genotoxicity and carcinogenic effects of PAHs in food. Their levels serve as indicators of PAH occurrence in food. The European Commission has set a maximum limit for these four PAHs (PAH4) at 10 μg/kg and for BaP at 2 μg/kg [31].

### 2.1. Formation Pathways

Previous literature has identified three main mechanisms of PAH formation in food: (1) pyrolysis of organic substances at temperatures above 200 °C, (2) adherence of PAHs-containing smoke to food when fat droplets fall on heat sources, and (3) incomplete combustion of fuel or charcoal, exposing food to released PAHs [14,32,33,34].

**Figure 1:**
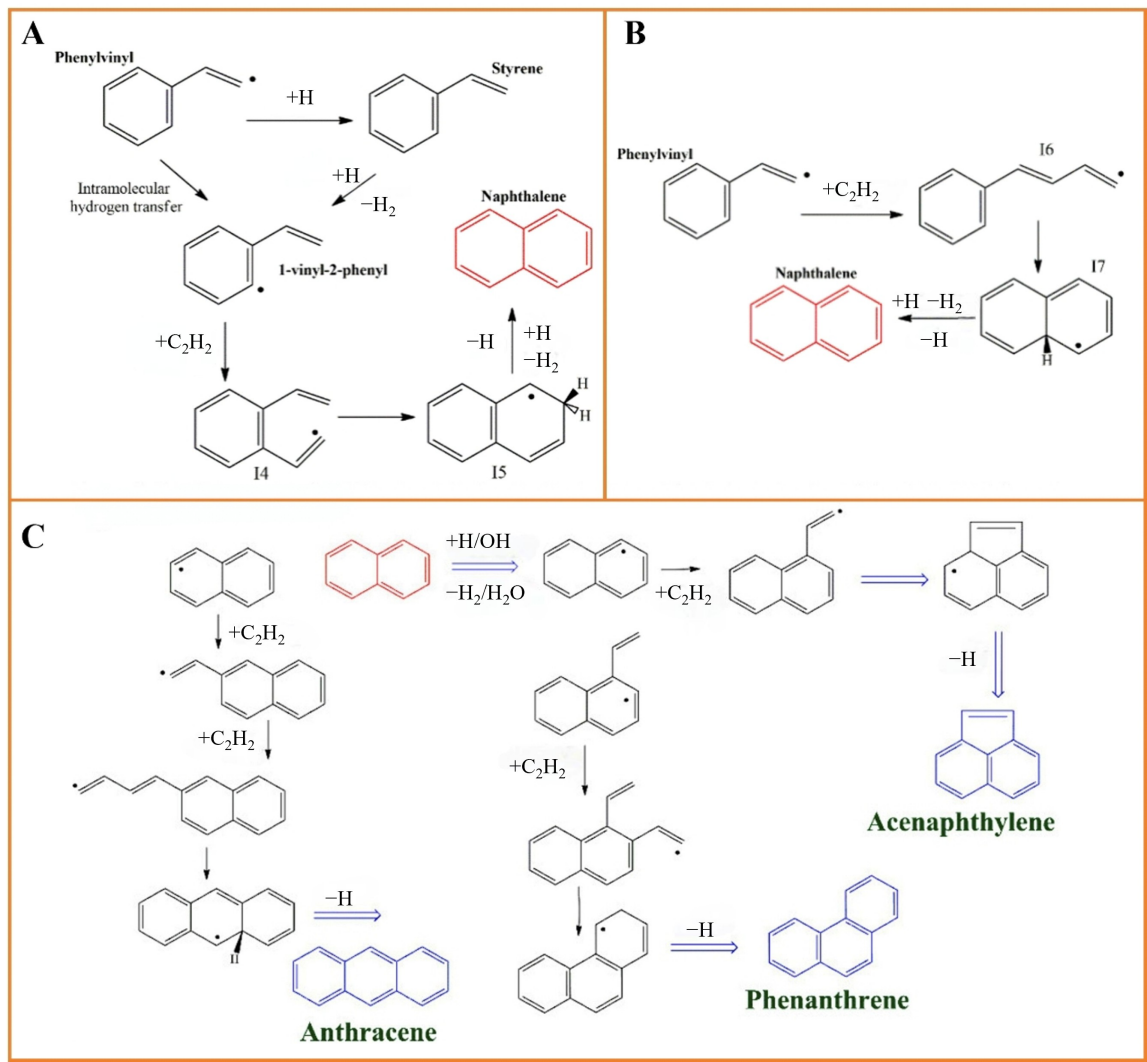
The generating mechanisms of PAHs. (**A**) Frenklach Mechanism. (**B**) Bittner-Howard Mechanism. (**C**) HACA Mechanism [36].

To date, the three main mechanisms for PAH formation are the Frenklach, Bittner-Howard, and H-Abstraction-C_2_H_2_-Addition (HACA) mechanisms [14,35]. These involve benzene ring stacking, radical polymerization, and chemical reactions. (1) Frenklach Mechanism: This mechanism rests on the formation of 1-vinyl-2-phenylradical either via intramolecular hydrogen transfer from the aromatic ring to the vinyl group (phenylvinyl → 1-vinyl-2-phenyl radical) or via H-abstraction from the orthosite in a styrene molecule. The addition of an acetylene molecule to the radical site in the 1-vinyl-2-phenyl radicals followed by ring cyclization (I4 → I5) and a loss of a hydrogen atom (I5 → naphthalene), typically operated by the H/C/O radical pool. (Figure 1A) [14]. (2) Bittner-Howard Mechanism: This pathway features the addition of an acetylene molecule to the side chain vinylic group in the phenylvinyl radical (phenylvivyl + C_2_H_2_ → I6) followed by a ring closure (I6 → I7) and a loss of a hydrogen atom (Figure 1B) [7]. (3) HACA Mechanism: Alkenes and alkynes generate benzene rings. Phenyl forms via dehydrogenation, and styrene forms via the HACA reaction. 2-Ethynyl-1-phenyl radical forms, leading to 2-naphthyl through HACA reaction with ethyne. This yields 2-ethynyl naphthalene and 2-ethynyl-3-naphthyl, which cyclize to form PAHs (Figure 1C) [36].

### 2.2. Influencing Factors

#### 2.2.1. Food Raw Materials

PAHs are persistent environmental pollutants ubiquitous in air, soil, and water. Consequently, crops like wheat, corn, peanuts, and beans absorb PAHs during growth, leading to potential accumulation [37]. Similarly, poultry and livestock exposed to PAHs-contaminated air, water, or feed can accumulate PAHs in their flesh [38].

#### 2.2.2. Food Composition

Fatty foods are more likely to produce PAHs during thermal processing because fats are more prone to incomplete combustion and pyrolysis reactions at high temperatures. Additionally, oils containing higher levels of unsaturated fats are more likely to produce smoke and PAH derivatives [39]. The content and proportion of components such as proteins and carbohydrates in food also affect the formation of PAHs. However, some trace components in food, such as antioxidants like vitamin C (V_C_) and vitamin E (V_E_), may have a certain inhibitory effect on the formation of PAHs [40].

#### 2.2.3. Processing Patterns

High temperatures promote incomplete combustion and pyrolysis, driving up PAH levels, with grilling, smoking, and frying being key culprits [41,42]. Direct flame contact, like in grilling and smoking, generates far more PAHs than indirect methods like boiling, microwaving, and baking due to higher temperatures and ample oxygen [40,43,44].

### 2.3. Tracing Approaches

Both liquid chromatography (LC) and gas chromatography (GC) can be used for qualitative and quantitative analysis of PAHs. LC systems often use a fluorescence detector (FLD) for high selectivity and sensitivity, with mass spectrometry (MS) due to its comparable sensitivity. Ultraviolet/Visible Light Detectors (UV-Vis) and Diode Array Detectors (DADS) have lower sensitivity. The limit of quantification for LC-FLD can reach ppb (μg/kg) or even ppt (ng/kg) levels, influenced by sample pre-enrichment, matrix/analyte combinations, and selected wavelengths for PAH analysis. Gradient elution is commonly used in LC methods, with acetonitrile as the organic phase and ultrapure water as the aqueous phase. A dedicated C18 column provides good separation for 16 U.S. EPA PAHs. However, separating isomers like P and BgP among the 16 EU PAHs remains challenging even with stepped elution gradients [45]. FLD requires different excitation and emission wavelengths for each PAH. In addition, PAHs that do not fluoresce, such as Flu, need to be detected using DADS. Ultra-performance liquid chromatography (UPLC) has been used to analyze PAHs [46,47]. GC coupled with MS is a common method for assessing PAHs due to its high sensitivity and selectivity [48].

Surface-enhanced Raman scattering (SERS) enhances the Raman signal of PAHs by metal nanoparticles, such as gold nanoparticles, to achieve highly sensitive detection [49,50]. In addition, enzyme-linked immunosorbent assay (ELISA) and electrochemical sensors are also suitable for the detection of PAHs [7,51,52]. Electrochemical sensors offer high sensitivity, ease of operation, and detection limits down to the nanomolar range (Table 1) [53,54,55,56,57]. Future research will focus on the development of new sensor materials and improving the stability and reusability of sensors to meet practical application needs.

### 2.4. Control Measures

The control measures for PAHs in food mainly include three aspects: (i) pre-thermal processing treatment of raw materials, such as selecting suitable fuels, using marinades, filtering/collecting juices/fats, and pre-heating; (ii) controlling thermal processing temperature and time, and optimizing equipment; and (iii) post-thermal processing treatment of finished products, like rinsing with hot water and using low/high-density polyethylene packaging.

#### 2.4.1. Mitigation Measures Prior to Thermal Processing

To reduce PAH formation, use clean fuels (e.g., natural gas) instead of wood/coal [32]. Pre-process raw materials by washing, cutting, and marinating with antioxidant-rich solutions. Effective marinades include honey-spice mixtures and natural herbs [62]. Adding bioactive compounds (e.g., V_C_ and V_E_) and low-unsaturation oils (e.g., palm oil) also helps. Filters with zeolite or activated carbon can reduce PAHs in smoked fish. Barriers between meat and smoke, preheating, or wrapping in aluminum foil can lower PAHs during grilling [35].

#### 2.4.2. Optimization of Thermal Processing Techniques

To reduce PAH exposure, avoid high temperatures and long cooking times. When frying, keep oil at 180 °C for a few minutes. Preheat meat in aluminum foil or use indirect grilling to prevent direct flame contact. Avoid reusing oil for high-heat cooking. Use temperature-controlled equipment with smoke filtration and efficient range hoods. Grilling with slate or nonstick cookware can also help.

#### 2.4.3. Reduction Measures After Thermal Processing

Mahugija et al. (2018) [63] showed that washing smoked fish with distilled water at 60 °C for 2–3 min effectively removed PAHs, reducing BaP levels from 390–1550 μg/kg to undetectable levels. Kuźmicz et al. [64] found that high-density and low-density polyethylene packaging significantly reduced PAHs in roast duck and smoked sausage, with BaP levels below 2 μg/kg. However, current PAH reduction strategies remain inadequate. Biodegradation using bacterial or fungal enzymes shows potential but is limited in food applications. For example, *E. coli* can degrade 58.22–71.04% of PAHs in oil distillates, and Lactobacillus bulgaricus can remove 60.50–76.80% of PAHs in smoked meats, relying on cell wall adsorption rather than cell viability [65,66,67]. Novel materials like cellulosic aerogels and biomass-derived nanocomposite films also show potential for PAH removal without compromising food quality, though further research is needed [68,69]. Overall, more efficient PAHs-removal technologies are essential to improve food safety.

## 3. Heterocyclic Aromatic Amines (HAAs)

HAAs can be divided into two categories based on their chemical structures: aminoimidazoleazaarenes (AIAs, or IQ-type HAAs) and aminocarbolines (ACs, or non-IQ-type HAAs). IQ-type HAAs, formed at temperatures between 100–300 °C, include quinoline, quinoxaline, pyridine, and furanopyridine classes and are polar. Non-IQ-type HAAs, formed above 300 °C through thermal decomposition of proteins or amino acids, include α-, β-, γ-, δ-carboline, and phenylpyridine classes and are non-polar. Among these, 2-amino-1-methyl-6-phenylimidazo[4,5-b] pyridine (PhIP) is the most commonly found and studied HAAs in foods [70,71,72].

### 3.1. Formation Pathways

HAAs mainly form via the Maillard reaction, with creatine (or creatinine), free amino acids, and sugars as key precursors [73]. Understanding their formation pathways is crucial for controlling their presence in food and studying their elimination. However, due to the vast number of HAA species and the complexity of their formation mechanisms, most pathways remain unclear. Current research is limited to a few common types of HAAs.

#### 3.1.1. Formation of Quinoxaline Heterocyclic Amines

The formation of quinoline and quinoxaline-type HAAs follows two main pathways [74]. The first involves creatine cyclizing and dehydrating to form creatinine above 100 °C. Sugars and amino acids then dehydrate and cyclize to form pyridine/pyrazine moieties, which condense with Strecker degradation aldehydes to produce imidazoquinoline and imidazoquinoxaline compounds. This pathway has been confirmed using carbon-14 labeling for the synthesis and identification of IQx, MeIQx, 4,8-DiMeIQx, and 7,8-DiMeIQx. The second pathway involves alkylpyridine or dialkylpyrazine radicals from the Maillard reaction reacting with creatinine to form quinoline and quinoxaline-type HAAs, though this pathway is still debated.

#### 3.1.2. Formation of Pyridine Heterocyclic Amines

PhIP, a common pyridine HAAs in thermally processed meats, forms primarily from phenylalanine and creatine (anhydride) via a pathway involving phenylacetaldehyde as a key intermediate [74,75]. The process, confirmed by carbon-13 labeling, includes Strecker degradation of phenylalanine to phenylacetaldehyde, which condenses with creatinine to form an unstable adduct. This adduct dehydrates and reacts further with phenylacetaldehyde, generating formaldehyde and ammonia, which ultimately form the imidazole ring of PhIP. Other amino acids like tyrosine, leucine, and isoleucine can also form PhIP when heated with creatinine.

#### 3.1.3. Formation of Aminocarboline Heterocyclic Amines

Aminocarbolines are non-polar HAAs in diets, formed mainly by high-temperature pyrolysis of proteins or amino acids. The pathways for Harman and Norharman (β-carbolines) are well understood, involving glucose and tryptophan as precursors. The process includes tryptophan’s Amadori rearrangement, dehydration, β-elimination, and intramolecular nucleophilic substitution, yielding β-carbolines. These compounds can be produced in large quantities by heating below 100 °C. Other studies suggest γ-carbolines form by heating creatinine, glucose, tryptophan, or isoleucine at 180 °C, α-carbolines from soy globulin pyrolysis, and δ-carbolines from glutamic acid pyrolysis. However, the specific formation pathways of these non-polar HAAs need further investigation.

### 3.2. Influencing Factors

#### 3.2.1. Precursor Substances

Many studies have shown that creatine, creatinine, amino acids, glucose, etc., are precursors for the production of HAAs. Different precursors play different roles. Sugars, creatine, and creatinine promote HAA formation, while a decrease in phenylalanine, serine, and leucine content can significantly increase HAA levels [76,77].

#### 3.2.2. Food Composition

The formation of HAAs in food is influenced by the content and ratio of proteins, fats, and carbohydrates, as well as amino acid composition. Foods rich in tryptophan, ornithine, and reducing sugars, as well as those with fat dripping during cooking, are more likely to produce HAAs through oil oxidation and heat conduction [70,78]. Conversely, maintaining moisture content during cooking can inhibit HAA formation [79].

#### 3.2.3. Processing Patterns

Higher temperatures and longer heating times generally increase HAA levels, though excessively long heating times can lead to HAA decomposition [77,80]. Acidic conditions reduce HAA formation, while alkaline conditions may promote it. Frying produces the most HAAs, followed by grilling and roasting, with boiling producing the least. Direct heat contact methods, like charcoal grilling, result in higher HAA levels. Controlling these factors is crucial to minimize HAAs in cooked foods.

#### 3.2.4. Exogenous Substances

Sugars, salt, and spices inhibit HAA formation, while soy sauce and cooking wine promote it. Common meat additives like textured protein, soy protein isolate, and starch may enhance HAA formation. Natural extracts (e.g., grape seed, rosemary, apple peel polyphenols) suppress HAAs by scavenging free radicals and capturing reactive intermediates [15]. Their effectiveness depends on structure, with meta-hydroxyl groups showing stronger inhibition [81].

### 3.3. Tracing Approaches

GC-MS is highly effective for analyzing low-polar or non-polar HAAs, but it requires derivatization to enhance the volatility of non-volatile HAAs, which can be time-consuming and risk contamination or errors from high injection temperatures. In contrast, HPLC is a reliable method for direct HAA detection without derivatization, though its sensitivity depends on detectors, which may face limitations like low sensitivity or interference from co-extracted substances. A common solution is HPLC coupled with tandem mass spectrometry (HPLC-MS/MS), which offers superior separation and precise qualitative and quantitative analysis of HAAs in complex food matrices [70]. Moreover, some HAAs can be quickly determined by measuring their absorbance or fluorescence intensity at specific wavelengths [82,83]. Electrochemical sensors with high sensitivity, low cost, and adaptability to complex food matrices are also expected to be further developed in the detection of HAAs (Table 2) [84,85,86,87,88].

### 3.4. Control Measures

#### 3.4.1. Control of Precursor Substances

Reducing sugars and creatine (or creatinine) or increasing certain amino acids can minimize HAA formation [7]. Adding histidine, leucine, methionine, and proline to beef patties can significantly inhibit HAA formation. Controlling moisture and fat content in meat processing can also reduce precursor migration and heat transfer, inhibiting HAA formation.

#### 3.4.2. Optimization of Processing Patterns

Avoiding high-temperature and long-duration cooking and choosing gentle processing techniques such as low-temperature simmering, vacuum cooking, and microwave heating can reduce the formation of HAAs [91]. Controlling the number of times oil is reused, as excessive reuse of oil can increase the content of HAAs [73]. Increasing the moisture content in food can also reduce HAAs formation, as water acts as a temperature regulator and prevents the migration of HAAs precursors through moisture evaporation. Furthermore, marinating meat with an acidic marinade (such as vinegar or lemon juice) before cooking can reduce HAAs formation, as the acidic environment may hinder the creation of certain HAAs [7].

#### 3.4.3. Addition of Exogenous Substances

Polyphenols and alkaloid compounds in herbal and tea extracts, as well as extracts from common spices, reduce the formation of HAAs by binding to precursors and scavenging free radicals [92]. In addition, hydrocolloids in powder or marinade form can inhibit the formation of HAAs in roast beef patties by interfering with the decarboxylation process in the Maillard reaction [93].

## 4. Furan

Furan is formed during the heat treatment of food and is commonly found in coffee, soy sauce, and canned foods. Coffee is the primary source of dietary furan exposure in adults. Unroasted green coffee beans contain little to no furan (approximately 4.1 μg/kg), while large amounts are produced during roasting (above 200 °C). Cereals are another significant source, contributing to 14% and 49% of total dietary furan exposure in infants and adolescents, respectively. Furan has been associated with liver damage in long-term studies. For instance, in a study where rats were exposed to doses of 0.44 mg/kg body weight (bw) and above, cholangiofibrosis emerged as an early and sensitive adverse effect, which significantly increased after 36 weeks. Furan exhibits hepatotoxicity in both rats and mice, with prominent effects including cholangiofibrosis in rats (at the highest dose of 8 mg/kg bw) and hepatocellular adenomas/carcinomas in mice (at doses of 4 mg/kg bw and higher) [94].

### 4.1. Formation Pathways

**Figure 2:**
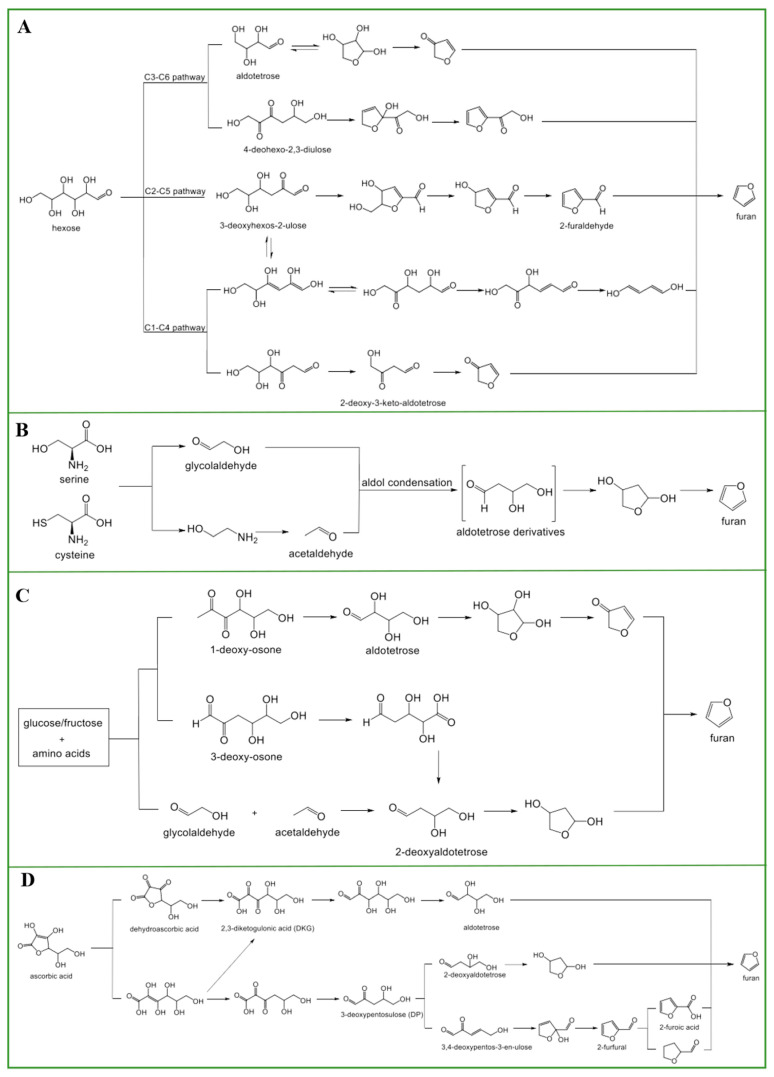
(**A**) Furan formation from hexose. (**B**) Furan formation from amino acids. (**C**) Furan formation via the Maillard reaction. (**D**) Furan formation from ascorbic acid.

Furan formation in food occurs through three main pathways: (1) thermal degradation of reducing sugars and amino acids; (2) Maillard reaction between reducing sugars and amino acids; and (3) thermal oxidation of ascorbic acid, polyunsaturated fatty acids, and carotenoids. Decarboxylation of 2-furfuric acid may also contribute [95,96] (Figure 2).

### 4.2. Influencing Factors

#### 4.2.1. Processing Patterns

High temperature accelerates the Maillard reaction and lipid oxidation, increasing furan formation [3,7]. Cooking methods, water content, and pH also have a significant effect on furan formation [3,97]. Acidic conditions promote fat oxidation and carbohydrate breakdown, while sugar degradation-related furan formation is lower at pH 4 and higher at pH ≥ 7.

#### 4.2.2. Precursor Substances

Carbohydrates, proteins, lipids, amino acids, sugars, ascorbic acid, and their derivatives are key furan precursors. Starchy foods and foods rich in reducing sugars are more prone to furan formation at high temperatures[98]. Similarly, foods high in unsaturated fatty acids, particularly α-linolenic acid and arachidonic acid, promote furan formation via oxidation intermediates like malondialdehyde. Additionally, ascorbic acid degradation during heat processing produces 4-deoxyascorbic acid, which undergoes hydrolysis, cyclization, and ring-opening to generate the furan precursor.

#### 4.2.3. Exogenous Substances

Divalent or trivalent metal ions like Cu^2+^, Cu^+^, and Fe^3+^ significantly promote furan formation from linoleic acid oxidation, with Cu^2+^ having the strongest effect, followed by Fe^3+^ and Cu^+^. In contrast, monovalent or divalent ions such as K^+^, Na^+^, and Ca^2+^ have no significant impact on furan formation. Polyphenolic compounds, including epigallocatechin gallate (ECG), myricetin, and quinic acid, can notably inhibit furan formation. Among them, myricetin shows the best inhibitory effect, with a maximum inhibition rate of up to 55.54%.

### 4.3. Tracing Approaches

Three main methods for detecting furan in food are solid-phase microextraction-gas chromatography-mass spectrometry (SPME-GC-MS), headspace injection-gas chromatography-mass spectrometry (HS-GC-MS), and headspace-solid-phase microextraction-gas chromatography-flame ionization (HS-SPME-GC-FID) [99,100,101,102]. HS-GC-MS is a well-established technique for isolating volatile furan from complex matrices, while SPME, known for its speed, solvent-free operation, selectivity, and sensitivity, is often preferred. Studies comparing HS and SPME in foods like coffee and baby food show both methods achieve satisfactory accuracy, but SPME offers higher sensitivity and is less affected by matrix interference [103,104]. In addition, GC-FID, GC-MS/MS, and electrochemical sensors have also been successfully applied to the detection of furans in food samples with good selectivity and sensitivity (Table 3) [105,106,107,108].

### 4.4. Control Measures

#### 4.4.1. Optimize the Processing Patterns

Lowering thermal processing temperatures effectively reduces furan formation without compromising food quality [97]. Microwave heating, due to its shorter duration, minimizes nutrient loss and furan formation [117,118]. Ohmic heating can slash furan content in baby food by 70–90%, while high-pressure processing at 5–20 °C both reduces furan and preserves food. High-pressure heat sterilization (90–120 °C) inactivates microbial spores, extends shelf life, and cuts furan formation by 81–96% in baby food purees [119,120,121].

#### 4.4.2. Control of Precursor Substances

Amino acids, sugars, polyunsaturated fatty acids, and ascorbic acid are key furan precursors, with varying furan formation from different precursors. Choosing raw materials with low furan precursors, such as increasing free amino acids in coffee beans and replacing sucrose/high fructose corn syrup in milk drinks with sugar alcohols, can reduce furan formation [15,97,122,123].

#### 4.4.3. Addition of Exogenous Substances

Adding natural antioxidants like V_C_, V_E_, and tea polyphenols can inhibit fat oxidation and free radical generation, reducing furan formation [3,15]. Synthetic antioxidants can also inhibit food oxidation and furan formation, but their usage must be carefully controlled for food safety. Enzyme preparations can break down furan precursors; for example, fructase converts fructose into less furan-prone sugars, while lipase promotes lipid hydrolysis, reducing furan formation [3]. In addition, Different mineral elements have different effects on furan formation: iron, magnesium, or low-concentration calcium ions promote it, while zinc and high-concentration calcium ions inhibit it.

#### 4.4.4. Control of Storage Conditions

Food should be kept in cool, dry, airtight places, and exposure time after opening should be minimized to reduce oxygen contact, thereby reducing furan formation [124]. Using packaging materials with good barrier properties, such as multilayer composite packaging materials, can effectively block oxygen and light, reducing oxidation during storage, extending shelf life, and reducing furan formation [7].

## 5. Acrylamide (AA)

Human exposure to AA comes from drinking water, tobacco smoke, and fried or baked foods like coffee and potato chips, which are associated with the Maillard reaction [125,126,127]. AA has strong tissue permeability and can be rapidly distributed in the blood and various tissues [128,129]. The European Union (EU) categorized AA as a secondary mutagen and carcinogen. In 2010, the European Chemicals Agency (ECA) added AA to its list of hazardous substances. In 2015, the EFSA confirmed that animal studies had shown AA and its metabolites to be damaging to deoxyribonucleic acid, thus establishing their genotoxic and carcinogenic properties [130].

### 5.1. Formation Pathways

#### 5.1.1. Asparagine Pathway (Asn)

**Figure 3:**
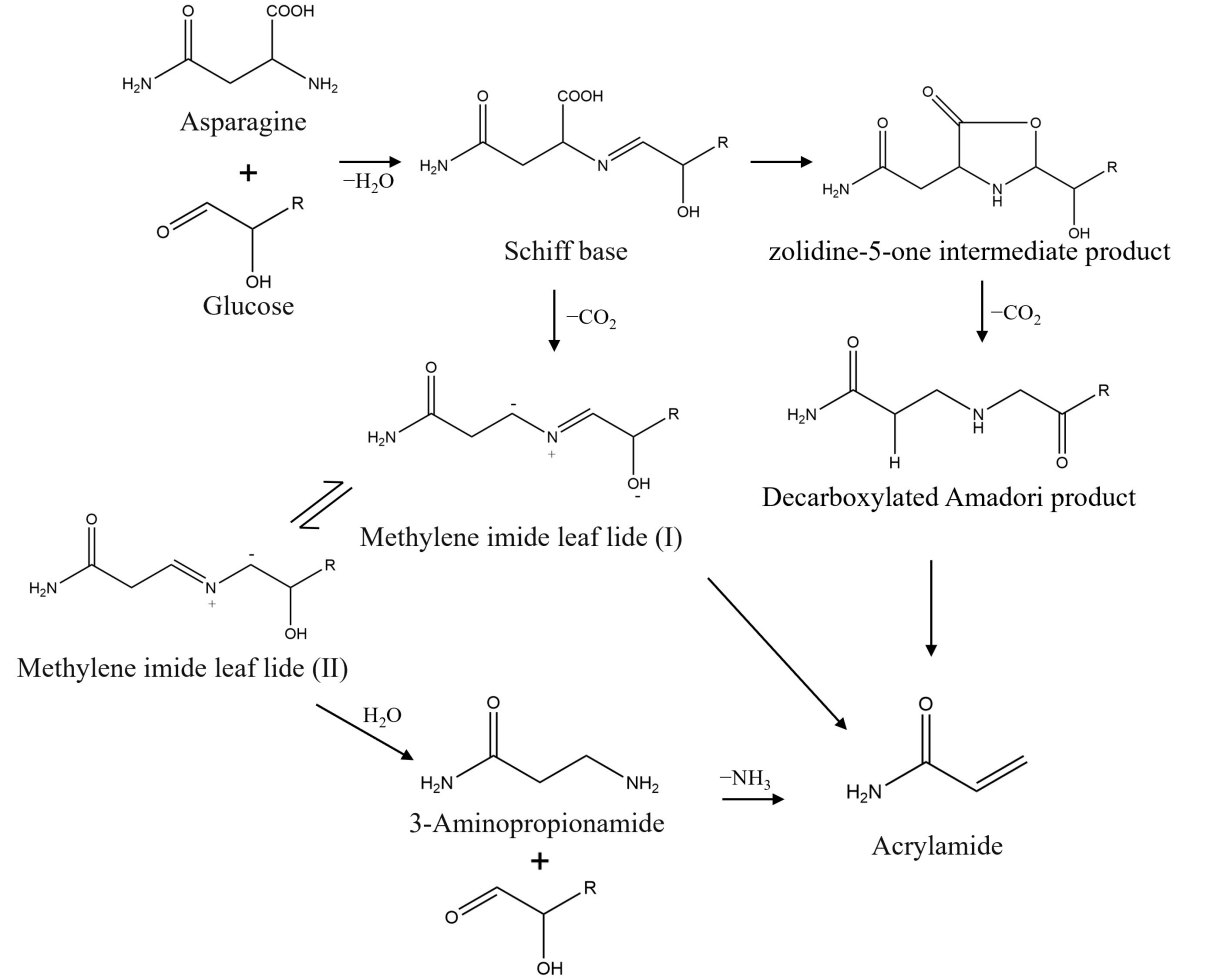
Proposed mechanism for the formation of AA during food processing between asparagine and reducing sugars.

The Maillard reaction, which can be divided into three stages, primarily produces AA in the first stage involving carbonyl-amine condensation and molecular rearrangement, where asparagine and reducing sugars react to form AA. At temperatures above 120 °C, they condense into Schiff bases, which degrade via Hoffmann’s elimination, leading to AA formation through two pathways [131,132] (Figure 3). (1) Intramolecular cyclization of the Schiff base forms a zolidine-5-one intermediate, which undergoes decarboxylation and rearranges into a decarboxylated Amadori product that converts to AA upon heating. (2) Schiff base decarboxylation produces methylene imide intermediates, which rearrange via 3-aminopropionamide deamination to yield AA.

#### 5.1.2. Non-Asparagine Pathways

AA can not only be converted from asparagine but can also be synthesized through other pathways. For example, AA can be obtained by reacting ammonia with acrolein or acrylic acid at high temperatures above 180 °C. In addition, the decomposition of oil at high temperatures will form substances such as triglycerides, which can also form acrylic acid or acrolein through oxidation, hydrolysis, and other reactions, and the product can react with ammonia to form a large amount of AA [133].

### 5.2. Influencing Factors

#### 5.2.1. Processing Patterns

Higher temperatures and longer processing times will increase AA content [131]. Pretreatments, including hot water blanching, can lower reducing sugars and asparagine, thus decreasing AA. Soaking potatoes in magnesium chloride and calcium chloride solutions reduced AA production by 74% and 67%, respectively. Additionally, pH influences AA formation, with acidic conditions (pH < 7) favoring the Maillard reaction and subsequent AA formation [7,134].

#### 5.2.2. Precursor Substances

Reducing sugars and asparagine are key precursors for AA formation, directly affecting the amount of AA produced. Asparagine has less impact than reducing sugars [97,122]. During bread baking, increased protein, fiber, and ash content provide precursors for AA formation. Amino acids from protein breakdown at high temperatures may contribute to AA formation. Fat content can change food’s thermal conductivity, impacting AA generation, with higher unsaturated fatty acid content leading to lower AA formation [122,135,136].

#### 5.2.3. Exogenous Substances

Food additives like sulfur dioxide from sodium bisulfite decomposition can inhibit AA formation [125,134]. Seaweed extracts and rice bran increase AA levels in bread. Conversely, green tea extract (GTE) and rosemary extract (RE) reduce AA, especially GTE [7,132,135]. L-asparaginase also reduces AA in food [129].

### 5.3. Tracing Approaches

LC-MS is widely used in food testing labs to directly analyze AA due to its high sensitivity and selectivity. For example, an optimized LC-MS/MS method accurately quantifies AA in chicory samples by pre-spiking AA and AA-d3 to create matrix-matched calibration curves [134,137,138,139]. Similarly, Feng et al. [73] used MSPE with HPLC-MS/MS to enrich AA using cysteine-functionalized covalent organic frameworks, achieving good linearity and low detection limits. Novel sensing platforms for in-situ colorimetric analysis and UV-Vis quantification have also been used to detect AA [131]. In addition, Fourier transform infrared spectroscopy (FTIR), fluorescence spectroscopy [17,18,140], and electrochemical sensors [141,142,143,144] also have greater prospects in monitoring AA (Table 4).

### 5.4. Control Measures

#### 5.4.1. Control Precursor Substances

Reducing the sugar and asparagine content is crucial for controlling AA formation. Choosing raw materials with lower levels of these precursors can reduce AA formation at the source. Soaking and blanching, such as using magnesium chloride and calcium chloride solutions for potatoes, can significantly reduce AA production [125].

#### 5.4.2. Optimize Processing Patterns

Low temperature and short-term processing are effective methods to control AA formation. Vacuum low-temperature frying, combining vacuum technology and frying dehydration, uses hot oil to process food at low temperatures and under negative pressure, promoting rapid water evaporation, shortening frying time, and significantly reducing AA generation [130,147]. Microwave frying technology can effectively reduce moisture content, frying temperature, and frying time, reducing AA content by 37–83% compared to traditional frying [148].

#### 5.4.3. Addition of Exogenous Substances

Several strategies can inhibit AA formation in food. Natural antioxidants, such as plant polyphenols, tomato extract, pomegranate extract, catechins, and other similar extracts, can prevent lipid oxidation and the formation of acrolein, thereby reducing AA levels [135,149,150]. Amino acids can inhibit AA formation by competing with asparagine or forming adducts; methionine reduces AA by interacting with it at 160 °C. Probiotics, especially lactic acid bacteria, adsorb AA and bind it to cell walls, while metal cations mitigate AA in high-temperature foods. Asparaginase reduces AA by converting asparagine into ammonia and aspartic acid or N-acetyl-L-asparagine, which is applicable in potato pre-treatment and baked goods. Hydrocolloids in various forms interfere with AA formation via functional groups, emerging as natural remedies [151].

## 6. Trans Fatty Acids (TFAs)

TFAs are unsaturated fatty acids with one or more independent, unconjugated double bonds in a trans configuration [152]. The main trans fatty acids in food are trans oleic acid, trans linoleic acid, and trans eicosatetraenoic acid [153,154,155]. Heating primarily induces the formation of trans-C18:2 and trans-C18:3 isomers. The EU regulations stipulate that the benchmark value of TFAs in general food products is 2% of the total fat content [156].

### 6.1. Formation Pathways

Unsaturated fatty acids in natural fats, primarily in the cis form, can convert into TFAs. TFAs are categorized into naturally occurring ones, found in small amounts in ruminant fats and products like meat and milk, and those formed during food processing through hydrogenation and heat treatment (e.g., refining vegetable oils and frying) [157]. Controlling TFA formation during food processing is essential to reduce their content, as research on the mechanisms of TFA formation, which vary between monounsaturated and polyunsaturated fatty acids, is still limited [158,159].

#### 6.1.1. Formation Mechanism of Monounsaturated Trans Fatty Acids

**Figure 4:**
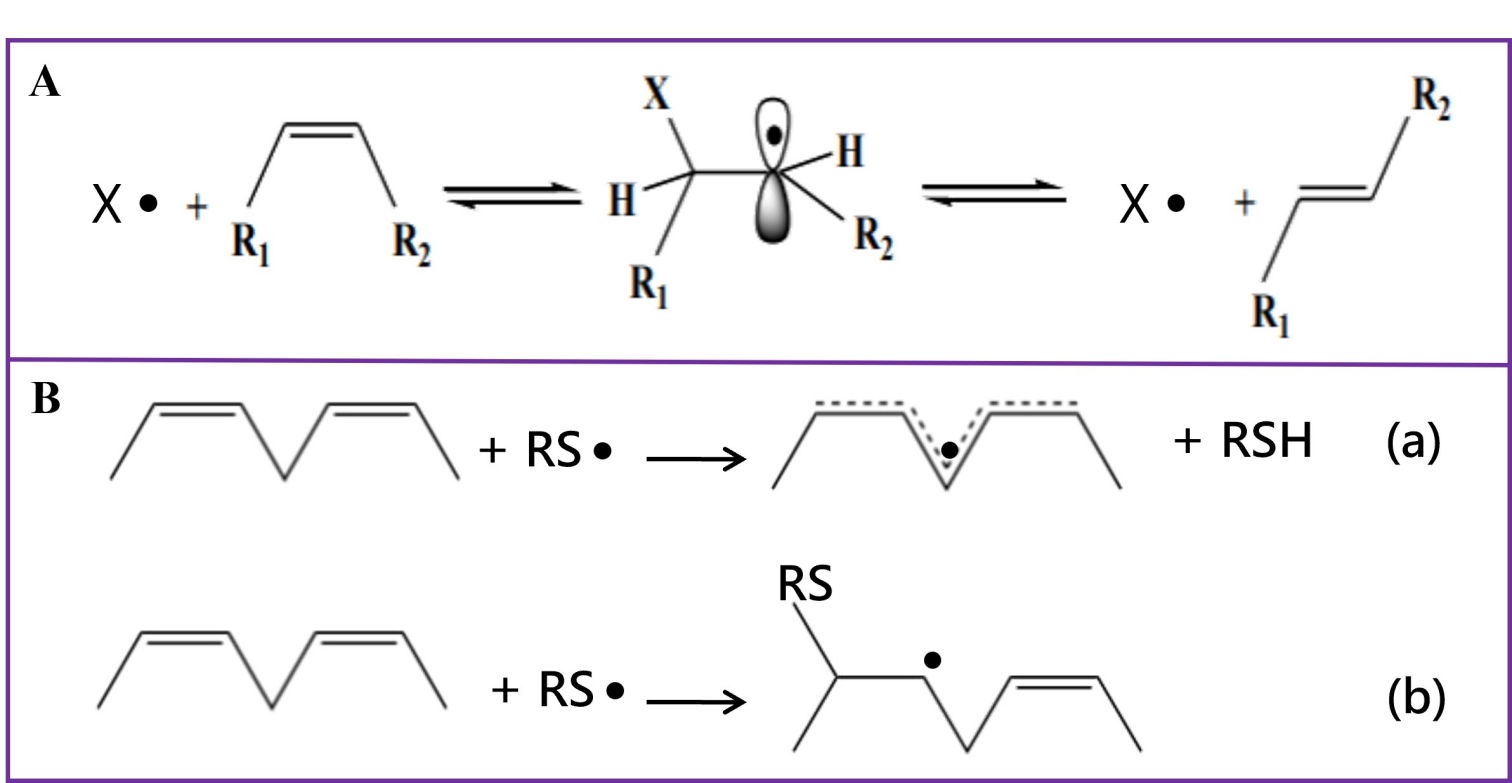
(**A**) Mechanism of formation of trans monounsaturated fatty acid formation. (**B**) Mechanism of formation of trans-polyunsaturated fatty acids. (**a**) Formation of pentadiene radicals. (**b**) Formation of adducts.

Monounsaturated trans fatty acids are formed via a free radical pathway. As shown in Figure 4A, monounsaturated trans fatty acids form via a free radical pathway. Light and heat catalyze the cleavage of unsaturated fatty acids into alkoxy radicals (R•). Free radicals (X•) react with R•, forming unstable intermediates. β-site removal then occurs, yielding a more thermodynamically stable, yet unnatural, trans isomer.

#### 6.1.2. Formation Mechanism of Polyunsaturated Trans Fatty Acids

Hydrogenation is one of the main pathways for the formation of trans fatty acids. Hydrogenation involves adding hydrogen gas to the double bonds of unsaturated fatty acids in the presence of a catalyst. For polyunsaturated fatty acids in vegetable oils, the formation pathways include both free radical mechanisms and intramolecular rearrangements (Figure 4B). In polyunsaturated trans fatty acids, only one of the double bonds undergoes isomerization to form the trans structure [7].

### 6.2. Influencing Factors

#### 6.2.1. Processing Patterns

Higher temperatures and longer heating increase TFAs [159,160,161]. Low-moisture environments boost TFA formation due to oil contact with the heating medium [7]. Frying container materials affect TFA formation; stainless steel is best due to its high stability and minimal TFA contribution [162].

#### 6.2.2. Precursor Substances

Foods rich in fats, proteins, and carbohydrates are more likely to form TFAs. Raw materials with high levels of unsaturated fatty acids are more likely to generate TFAs during processing [163,164]. Free fatty acids may promote TFA formation [165]. The composition of cis fatty acids in plant oils affects TFA formation, with higher amounts of certain cis fatty acids leading to higher TFA content.

#### 6.2.3. Exogenous Substances

Antioxidants like α-tocopherol and BHT can inhibit TFA formation by suppressing isothiocyanate-induced UFA isomerization but are less effective against polysulfide—induced isomerization [160,162,166]. Isothiocyanates and polysulfides, which are sulfur-containing compounds, significantly promote the thermal isomerization of unsaturated fatty acids, increasing TFA formation [159].

### 6.3. Tracing Approaches

GC is a common method for detecting TFAs, effectively separating them from food samples and converting them into volatile fatty acid methyl ester derivatives for analysis with FID or MS detectors [163,167]. GC-FID analyzes cis/trans isomers and calculates trans isomer ratios, while GC-MS can detect sterol oxides in vegetable oils, indicating oxidation and TFA formation. For thermally unstable samples, HPLC with UV or ELSD detectors offers high selectivity and accuracy, and coupled with MS, it can identify positional isomers of TFAs. NMR provides accurate TFA identification and quantification without derivatization, though its high cost limits its use in research and high-end testing [168]. Spectroscopic methods like NIR and FTIR offer rapid, non-destructive TFA prediction and detection, with NIR suitable for online food production monitoring and FTIR measuring specific peaks associated with trans bonds (Table 5) [169,170,171].

### 6.4. Control Measures

Lowering the cooking temperature and shortening the cooking time can reduce UFA isomerization [184]. Avoid reusing frying oil to reduce TFA generation. Choosing the right frying vessel (e.g., stainless steel) also reduces TFA formation [42,162]. UFAs isomerization can be reduced by using oils rich in natural saturated fatty acids (e.g., coconut, palm), non-hydrogenated, fully hydrogenated, or phytosterol esters [159]. Natural antioxidants such as apple pomace and olive leaf extracts can inhibit the formation of TFAs [158,185,186]. Synthetic antioxidants such as TBHQ and BHT can also effectively inhibit the oxidation and isomerization of oils, reducing the formation of TFAs [7].

## 7. Advanced Glycation End-Products (AGEs)

AGEs are harmful products formed by the reaction between amino groups of proteins or nucleic acids and carbonyl groups of reducing sugars through non-enzymatic browning [187]. Key AGEs include Nε-carboxymethyl-lysine (CML), Nε-carboxyethyl-lysine (CEL), pyrraline, and pentosidine, with CML and CEL being the most studied [188]. AGEs can be divided into endogenous AGEs (glycosylation of reducing sugar and protein in living organisms) and exogenous AGEs (ingested from food) [189]. AGEs can also be classified by molecular weight (low and high), properties (fluorescent and crosslinking, non-fluorescent and non-crosslinking), and types of dicarbonyl compounds (e.g., glyoxal-AGEs and methylglyoxal-AGEs) [7,190]. Exogenous AGEs have been identified as ongoing risk factors for human health. However, recent studies have shown that small amounts of AGEs may have little or no effect on humans [191]. Despite this, there are currently no laws or regulations that clearly define the acceptable levels of AGEs in food. Excess AGEs in the body can trigger oxidative stress and inflammation. These processes contribute to aging and various chronic diseases such as diabetes [192], Alzheimer’s disease [193], atherosclerosis, and uremia [194].

### 7.1. Formation Pathways

**Figure 5:**
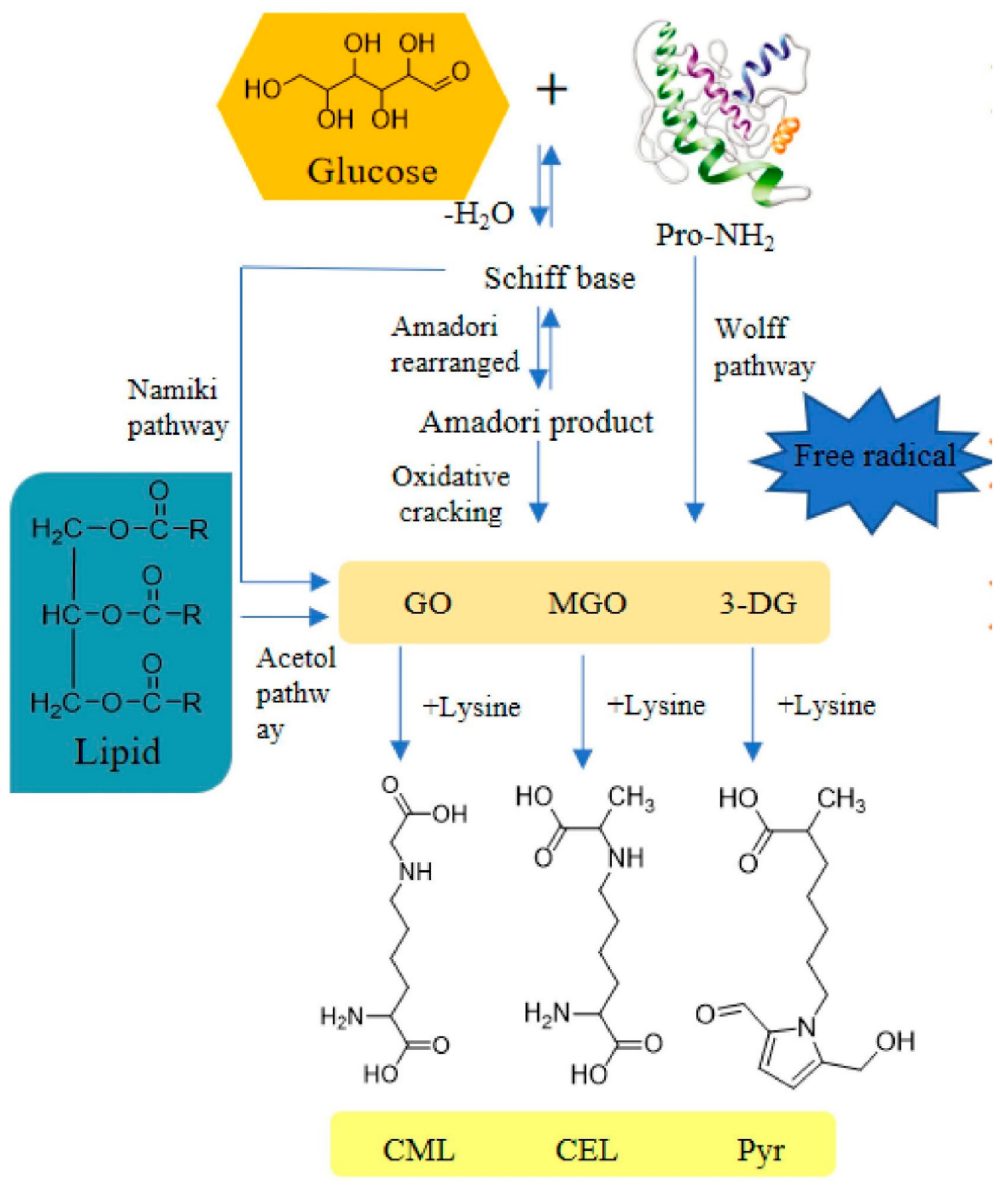
Formation mechanism of AGEs in food thermal processing [3].

AGEs can be formed through a number of reaction pathways (as shown in Figure 5), the most classic of which is the Maillard reaction pathway [195,196,197]. It consists of three stages: In the first, the free amino and carbonyl groups react to form an unstable Schiff base, which then rearranges into a more stable Amadori product. In the second, Amadori products undergo enolation to form dicarbonyl compounds like glyoxal (GO) and methylglyoxal (MGO), intermediates for CML and CEL formation. In the final stage, these dicarbonyl compounds undergo Stlerker degradation with protein amino groups, forming stable, irreversible AGEs. AGEs can also be generated through pathways other than the Maillard reaction [198,199]. (1) Acetol pathway: Fat oxidation forms dicarbonyl compounds that react with lysine to create AGEs. (2) Hodge pathway: Amadori products oxidatively crack to form AGEs. (3) Namiki pathway: Schiff bases oxidize to produce dicarbonyl compounds and AGEs. (4) Wolff pathway: Reduced sugars degrade via metal-catalyzed oxidation to form dicarbonyl compounds, leading to AGEs through Stellwag degradation. (5) Dunn pathway: Ascorbic acid reacts with lysine to form AGEs under suitable conditions.

### 7.2. Influencing Factors

#### 7.2.1. Precursor Substances

AGE formation in food is influenced by precursors like sugars, proteins, and lipids. Reducing sugars, which start the Maillard reaction, increase AGE risk when present in higher amounts. Amino acids, peptides, and proteins can react with α-dicarbonyl compounds to form AGEs. High-protein foods, especially beef, tend to form more AGEs than pork and chicken. Lysine and arginine residues in proteins also affect the formation of AGEs. However, high-carbohydrate foods tend to form fewer AGEs [188,200,201,202].

#### 7.2.2. Processing Patterns

High temperatures and low moisture (e.g., baking, frying) accelerate AGE formation through sugar-protein reactions, while low-temperature, humid methods (e.g., steaming, boiling) produce fewer AGEs [203,204,205]. Longer heating times and traditional methods like frying and grilling increase AGE content, whereas emerging technologies (e.g., air frying, high-pressure processing) can reduce it. The impact of microwave heating is debated, with some studies suggesting it accelerates the Maillard reaction [199,206,207]. Acidic conditions inhibit early Maillard reactions but may later promote AGEs, while alkaline conditions enhance certain AGEs [208].

#### 7.2.3. Storage Conditions

High temperature and prolonged storage can increase the levels of AGEs in meat products [4,195]. Preservation methods such as curing, drying, freezing, vacuum packaging, and smoking can affect the formation of AGEs. For example, sausages that undergo natural dehydration have lower levels of CML and CEL than those dried at medium or high temperatures.

#### 7.2.4. Exogenous Substances

Antioxidants like betanin inhibit AGE formation by trapping MGO and interacting with proteins [209]. Betanin’s thermal degradation products, like betalamic acid, also bind to proteins, further reducing AGE formation. Spices, especially flavonoids, inhibit AGE formation by trapping reactive carbonyl compounds and scavenging free radicals. In contrast, adding sugar and salt, such as in marinades like soy sauce or ketchup, increases AGEs precursors and AGEs like CML and pentosidine [190]. In addition, metal ions such as copper and iron can catalyze the AGE formation reaction [210].

### 7.3. Tracing Approaches

Fluorescence analysis is easy to perform but can only detect substances with fluorescent properties, such as crosslines and pentosidine, while most AGEs, like CML, CEL, and pyrraline, do not fluoresce. ELISA, based on the antibody-enzyme complex principle with color development, is a convenient and rapid method often used to determine AGEs [211]. However, ELISA results are typically expressed in kU/100 g or kU/100 mL, which cannot be converted to the common AGE units. ELISA also requires specific antibodies for each compound and is prone to matrix interference, limiting its application.

Chromatography methods, including HPLC, LC-MS/MS, and GC-MS, offer higher sensitivity and accuracy for AGE detection [212,213,214,215]. GC-MS can measure CML in meat but requires derivatization and often detects lower CML levels in high-fat samples. LC-MS/MS is widely used for AGE analysis in food due to its efficiency, reproducibility, and lack of need for pre-column derivatization [216,217]. Electrochemical sensors have promising application prospects in the detection of AGEs due to their low cost and short measurement time (Table 6) [218,219,220,221,222].

### 7.4. Control Measures

#### 7.4.1. Control Precursor Substances

To minimize the formation of AGEs in food, it is crucial to use fresh and high-quality ingredients that are rich in active components and nutrients and have fewer AGE precursors. Additionally, ensuring the purity and quality of proteins and carbohydrates helps minimize adverse reactions caused by impurities, further reducing AGE formation [234,235].

#### 7.4.2. Optimize Processing Patterns

During processing, the amount of AGEs can be reduced by keeping the temperature as low as possible and shortening the time [236]. Technologies like ohmic heating, vacuum frying, high-pressure processing, and pulsed electric field treatment can reduce AGE formation by lowering processing temperatures and times [121,237,238]. Delaying the addition of soy sauce, particularly later in the cooking process, can also reduce AGE formation by shortening the exposure time of AGE precursors. Maintaining a lower pH in food helps reduce AGE formation [239]. Blanching as a pre-treatment can effectively reduce the levels of AGEs.

#### 7.4.3. Addition of Exogenous Substances

Antioxidants, polyphenols, vegetable extracts, and spices are effective in reducing AGE formation [240,241]. These compounds inhibit the oxidative degradation of Amadori products, scavenge free radicals, chelate transition metals, and neutralize carbonyl intermediates. Epigallocatechin gallate (EGCG) in black chokeberry has inhibition rates of 84.47% and 54.44% against α-dicarbonyl compounds and AGEs, respectively [242]. Similarly, betanin, a natural antioxidant, reduces AGE formation, and its encapsulation in CS@QAMSNPs enhances stability and effectiveness in high-temperature environments while adsorbing existing AGEs. This nanomaterial not only controls AGE formation in food but also reduces their absorption during digestion [243]. Peptides, particularly myostatin, prevent early glycation and reduce late-stage AGEs [239]. Certain amine compounds, such as lysine, competitively react with reducing sugars to lower AGE formation. Moreover, citric and acetic acids reduce CML and CEL levels in pork by inhibiting the Maillard reaction [198].

#### 7.4.4. Control of Storage Conditions

Low-temperature and low-humidity storage conditions can slow down the formation of AGEs. Use packaging materials that can reduce the oxidation of food during storage, thereby minimizing the accumulation of AGEs [199].

## 8. Sterol Oxides

**Figure 6:**
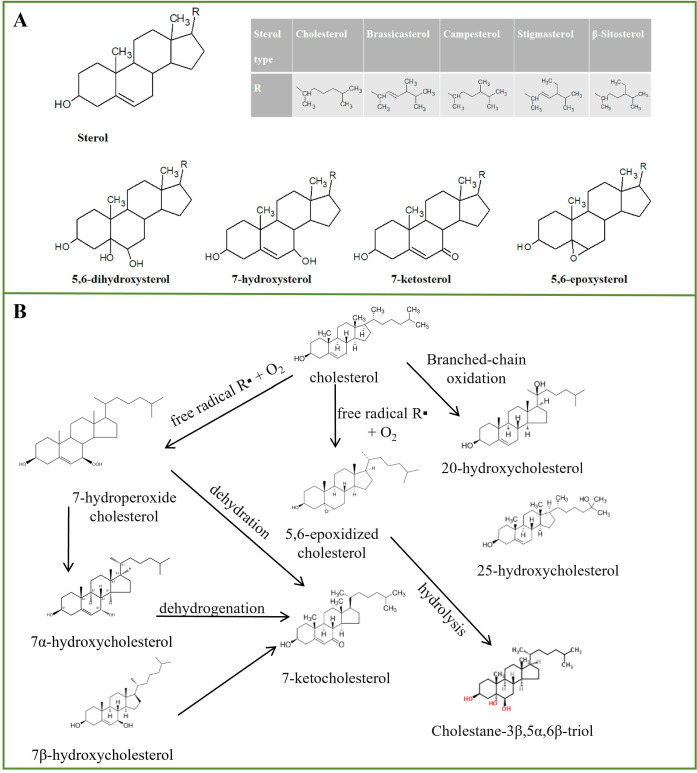
(**A**) General structure of sterols and their oxidized derivative. The table shows common phytosterol structures along with cholesterol. Polar sterol oxidation products are shown: 5,6-dihydroxysterol, 7-hydroxysterol, 7-ketosterol, and 5,6-epoxysterol [248]. (**B**) The formation process of cholesterol oxide.

Sterols, essential cell membrane components in animals and plants, are widely found in daily diets like meat, eggs, and oils [244,245]. They include animal-derived cholesterol and plant-derived phytosterols (PS), such as β-sitosterol, campesterol, and stigmasterol (Figure 6A). During food production, processing, and storage, sterols can oxidize and degrade due to light, heat, oxygen, and metal ions, forming cholesterol oxidation products (COPs) and phytosterol oxidation products (POPs). COPs are common in thermally processed meats, while POPs are prevalent in chips, fries, baked goods, and PS-fortified foods. Typically, oxidation levels of COPs and POPs are 0.1–1%, but in fried meat, COPs can reach 6–10% [246,247].

### 8.1. Formation Pathways

#### 8.1.1. COPs Formation Pathways

In food products, endogenous COPs form through non-enzymatic oxidation or autoxidation, while exogenous COPs arise from cholesterol oxidation reactions triggered by external factors like enzymes, chemicals, photochemistry, and radiation (Figure 6B). During thermal oxidation, compounds such as 7α-hydroxycholesterol (7α-OH), 7β-hydroxycholesterol (7β-OH), and 7-ketocholesterol (7-keto) are primarily generated by oxidation at the C-7 position. Thermal processing methods like frying, grilling, or microwaving can oxidize the double bonds in cholesterol molecules, producing various COPs, including 7-ketocholesterol, 7α/β-hydroxycholesterol, and α/β-epoxycholesterol [244,245]. Photo-oxidation involves cholesterol reacting with triplet and singlet oxygen to form air-oxidized COPs such as 5α,6α-epoxycholesterol (5α,6α-EP) and 5β,6β-epoxycholesterol (5β,6β-EP), which can further hydrolyze into toxic cholestane-3β,5α,6β-triol (triol). Oxidation at C20 and C25 in the cholesterol side chain produces 20-hydroxycholesterol and 25-hydroxycholesterol, respectively. Light exposure due to non-light-proof packaging can trigger cholesterol oxidation in cholesterol-containing foods [249]. Additionally, metal ions such as iron and copper in food can catalyze cholesterol oxidation, promoting COP formation. Using iron or copper containers to process cholesterol-containing foods may increase COP formation [244,250].

#### 8.1.2. POPs Formation Pathways

**Figure 7:**
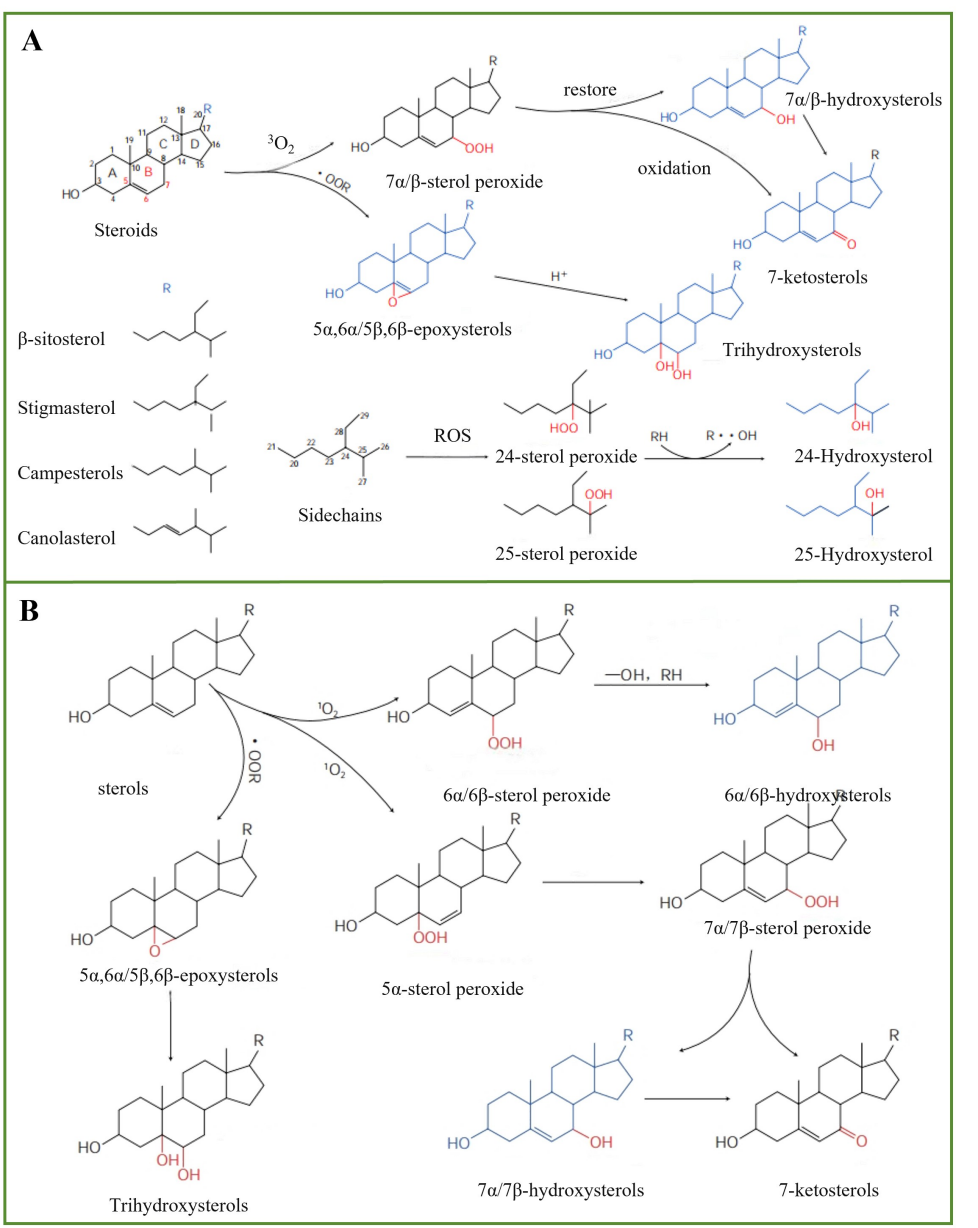
(**A**) Automatic oxidation mechanism of PS. (**B**) Photosensitized oxidation pathways of PS.

Phytosterol oxidation, similar to cholesterol, involves enzymatic and non-enzymatic pathways. Enzymatic oxidation occurs within the body, while non-enzymatic oxidation happens outside. Research on phytosterol oxidation and POPs focuses on autoxidation, thermal oxidation, and photosensitized oxidation in food systems. (1) Autoxidation: Initiated by triplet oxygen, phytosterol autoxidation is a free radical chain reaction that generates primary products like hydroperoxides, which then form stable secondary products such as 7-keto, 7α/7β-hydroxy, 5α,6α-epoxy, 5β,6β-epoxy sterols, and 24-hydroxy sterols (Figure 7A). These are commonly analyzed in food testing. (2) Thermal oxidation: Similar to autoxidation initially, phytosterol thermal oxidation accelerates at high temperatures and forms polymerization products like dimers, oligomers, and polymers during later stages [251]. (3) Photosensitized oxidation: Photosensitizers like chlorophyll and riboflavin in food absorb light and excite triplet oxygen to form singlet oxygen, which reacts with phytosterols containing double bonds. This process generates free radicals added to the C5 and C6 atoms of the sterol nucleus, triggering C7 site reactions. Under natural or artificial light, it mainly produces 6β-hydroxy, 7α-hydroxy, and 7β-hydroxy sterols [252]. Photosensitizers promote this oxidation, and higher light intensity increases the oxidation rate constant, leading to faster oxidation (Figure 7B) [253,254].

### 8.2. Influencing Factors

#### 8.2.1. Processing Patterns

Temperature significantly impacts the formation of sterol oxidation products. Prolonged heating at 180 °C for 8 h notably boosts plant sterol oxidation products like 7-ketosterols and 7-hydroxysterols [3]. Cooking methods also play a role. Microwave treatment can release phytosterols and enhance their stability by freeing antioxidants like phenolic compounds and tocopherols, though excessive treatment may still cause oxidation [255]. Lower pH levels, such as in a saline environment, can promote cholesterol oxidation by reducing protein interactions and exposing lipids to pro-oxidants [256].

#### 8.2.2. Food Components

The oxidation stability of PS depends on its structure, the oil type, and the surrounding matrix. For example, rapeseed sterols oxidize more easily than β-sitosterol. Additionally, refined oils, which lack antioxidants, are more prone to oxidation than cold-pressed oils [253]. PS oxidation is influenced by sterol type, lipid unsaturation, and metal ions, with stability generally following β-sitosterol > campesterol > stigmasterol. Lipid matrices with unsaturated bonds and certain food matrices help maintain PS stability [254]. Antioxidants inhibit PS oxidation, while pro-oxidants and metal ions accelerate it [244]. High-fat foods with unsaturated fatty acids are more likely to form COPs during processing, whereas saturated fats create a more stable environment [257]. In high-temperature conditions, sterols are more stable within unsaturated systems, whereas at low temperatures, they exhibit greater stability in saturated systems [258].

#### 8.2.3. Environmental Factors

Sterol oxidation is accelerated by high oxygen concentrations, increased air exposure, and larger oil-air contact areas, as seen in cut produce and fried foods. UV light and high temperatures also promote oxidation by breaking sterol bonds and degrading phytosterols [253,255]. Studies have shown that the content of phytosterol oxidation products increased by 0.3 mg/100 g after 35 days of storage at room temperature [259].

#### 8.2.4. Exogenous Substances

Natural antioxidants (e.g., fruit extracts) and synthetic antioxidants (e.g., tocopherols) reduce sterol oxide by neutralizing free radicals and stabilizing reactive oxygen species. However, metal ions like iron and copper catalyze oxidation, increasing sterol oxide with higher levels in food [251]. Salt in brine acts as a pro-oxidant, releasing iron ions and inhibiting antioxidant enzymes, which increases sterol oxide formation [256].

### 8.3. Tracing Approaches

Detecting sterol oxides in food currently lacks a unified standard. Given the similarity between plant sterols and cholesterol, many researchers use cholesterol oxide detection methods, which have proven effective. Modern instrumental analysis, such as HPLC, LC-MS/MS, GC, and GC-MS/MS, is the mainstream approach [248,260,261,262]. Lukáš Kolarič et al. [263] used two different analytical methods (HPLC and spectrophotometry) to determine cholesterol content in milk, with LODs and LOQs of 2.13 mg/kg and 6.45 mg/kg for HPLC and 12.55 mg/kg and 38.04 mg/kg for spectrophotometry, respectively. Both methods are suitable for the determination of cholesterol content in milk, but HPLC methods exhibit higher sensitivity and lower limits of detection or quantification, respectively. Hsu et al. [260] used GC-MS to analyze COPs in fried chicken fiber. Seven COPs were produced in fried chicken fiber, with the highest level of 35.220 μg/g, with 7α-OH and 7β-OH dominating COPs.

### 8.4. Control Measures

#### 8.4.1. Selection of Appropriate Raw Material

Selecting low-fat, low-cholesterol ingredients, using ingredients that have been defatted, or choosing ingredients rich in antioxidant components, such as nuts rich in V_E_, can protect cholesterol from oxidation and thus reduce the formation of sterol oxide [3].

#### 8.4.2. Optimize Processing Patterns

Avoiding high temperatures and prolonged heating can reduce the oxidation of sterols. For microwaving, reduce the power or shorten the time. Use stainless steel or glass to prevent metal ion release. If ingredients contain metal ions, reduce their content.

#### 8.4.3. Addition of Exogenous Substances

Parsley extracts, rich in flavonoids, can significantly reduce sterol oxide in fried eggs, with the best effect observed at a 0.75% parsley addition [244]. This highlights the efficacy of natural antioxidants in controlling cholesterol oxidation during food processing. Synthetic antioxidants such as BHT (Butylated Hydroxytoluene) and BHA (Butylated Hydroxyanisole) can also capture free radicals and interrupt oxidation chains, protecting cholesterol from oxidation. Poudel et al. [248] showed that combining liposomes with tocopherols significantly reduces sterol oxide, with the lowest sterol oxide content observed after microwave treatment. This underscores the potential of antioxidant combinations in mitigating sterol oxidation.

#### 8.4.4. Control of Storage Conditions

Using packaging materials that block oxygen and light, such as vacuum packaging and aluminum foil packaging, and storing food under low-temperature conditions can reduce the formation of sterol oxidation products [245]. Packaging materials containing antioxidants can slowly release antioxidants to continuously protect the cholesterol and other nutrients in food, reducing the formation of sterol oxide [251].

## 9. Conclusions and Outlook

Thermal processing is essential in food processing but can generate harmful substances linked to cancer and chronic diseases. Understanding the formation pathways, influencing factors, detection methods, and control measures of these contaminants is vital for minimizing their presence and ensuring food safety. Mitigation strategies include selecting fresh ingredients, optimizing cooking parameters, and adding antioxidants. In recent years, some modern, advanced, and novel processing technologies such as ultrasound (US), pulsed electric field (PEF), and high-pressure pretreatment (HHP), among others, have been used to reduce microbiological risks and improve organoleptic, nutritional, and functional properties in food, in addition to being environmentally friendly [264]. Some studies have also shown that these processing techniques have a potential inhibitory effect on the formation of contaminants in the thermal processing of food, so the use of new technologies to process food is a promising alternative to reduce the amounts of contaminants in thermal processing. It can be inferred that the use of non-thermal technologies promotes the generation of fewer thermal process contaminants due to the lower temperatures at which they are applied and the shorter processing time. New thermal technologies, such as US and PET, provide fast and uniform heating and reduce processing times, which are essential to avoid reactions that lead to the formation of hot-process contaminants. On the other hand, with regard to microwave heating, the results are controversial and further research is needed to understand the underlying mechanisms for the application of this technology. Studying the different conditions associated with new technologies and comparing them with conventional technologies is essential to better understand their impact on the formation of thermal process contaminants. In addition, there is great potential to detect these contaminants using convenient and low-cost sensors, such as electrochemical sensors, surface-enhanced Raman spectroscopy, etc. This will help develop safer food processing methods that maintain food quality while minimizing health risks. Table 7 describes the main emerging technologies and discusses their impact on the formation of thermal process contaminants.

## Figures and Tables

**Table 1:** List of analysis methods for PAHs in different food types.

Food Type	Analytical Techniques	Validation Parameters	References
Smoked bacon	RP-HPLC-(+) ESI/MS	LOD: 0.1–0.25 μg/kg LOQ: 0.50 μg/kg Recovery: 74–100%	[58]
Tea leaves	UPLC	LOD: 1.69–9.97 ng/kg LOQ: 5.12–30.21 ng/kg Recovery: 84.8–105.4%	[47]
Tea and coffee	MSPE-GC–MS/MS	LOD: 0.1–0.3 ng/L Recovery: 84.5–112.6%	[48]
Shrimp	Surface-enhanced Raman scattering (SERS) sensor (AuNPs)	LOD: 0.12 ng/mL RSD: 6.21%	[49]
Chicken	SERS sensor based on gold nanostars@reduced graphene oxide (AuNS@rGO)	LOD: 0.0028 μg/L Recovery: 89.2–100.8% RSD: 2.42–6.53%	[50]
Waters	Electroanalytical method on molecular imprinted polymer-glassy carbon electrode (MIP-GCE)	LOD: 12 nM LOQ: 40 nM	[59]
Waters	Novel imprinted polymer nanofilm sensor	LOD: 0.001 ng/L LOQ: 0.01–0.1 μg/kg Recovery: 83–110%	[60]
Waters	A novel hybrid plasmonic platform based on the synergetic combination of a molecularly imprinted polymer (MIP) thin film with Au nanoparticle (NPs) assemblies	LOD: 1 nM Recovery: 83–110%	[61]
Milk	SPME-GC-FID	LOD: 0.003–0.020 ng/mL Recovery: 92.15–106.64%	[37]

**Table 2:** List of analysis methods for HAAs in different food types.

Food Type	Analytical Techniques	Validation Parameters	References
Freeze-dried pork and pork jerky	Reversed-phase (RP)-UPLC-MS/MS	LOD: 0.005–0.05 μg/kg LOQ: 0.01–0.1 μg/kg Recovery: 59.4–104%	[89]
Pork patties	Fluorescence method	LOD: 0.224 μg/kg Recovery: 87.6–97.8%	[82]
Cantonese mooncake	MSPE-UPLC-MS	LOD: 0.01–7.01 ng/g Recovery: 62.12–126.86%	[76]
Fried chicken drumsticks	HPLC-MS		[8]
Roast beef	HAAs prediction model based on genetic algorithm and support vector regression	LOD: 0.028–0.214 ng/g LOQ: 0.097–0.625 ng/g Recovery: 101.8–105.6%	[77]
Roasted and pan- fried pork, and beef patties	HPLC-quadrupole-orbitrap HRMS	LOD: 0.02–0.6 μg/kg LOQ: 0.05–2.0 μg/kg Recovery: 71.3–114.8%	[90]
Roasted pork, fried chicken, etc.	HPLC-MS/MS	LOD: 0.020–0.375 μg/kg Recovery: 82.0–109.5%	[70]
Bread, cakes, and French fries	MSPE-HPLC-MS/MS	LOD: 0.012–0.210 μg/kg LOQ: 0.043–0.650 μg/kg Recovery: 90.4–102.8%	[73]

**Table 3:** List of analysis methods for furan in different food types.

Food Type	Analytical Techniques	Validation Parameters	References
Coffee	GC-MS	LOQ: 5 μg/kg Recovery: 80–110%	[103]
Semi-solid and paste-type foods	HS–SPME–GC/MS	LOD: 0.18 μg/kg LOQ: 0.54 μg/kg	[109]
Eight food matrices	HS–SPME–GC/MS	LOD: 0.01–0.02 μg/kg LOQ: 0.04–0.06 μg/kg	[110]
Various food samples	HS–SPME–Arrow GC–MS/MS	LOD: 0.001–1.071 μg/kg LOQ: 0.003–3.571 μg/kg	[105]
Thermally processed Mopane worms, corn, and peanuts	HS–SPME–GC/FID	LOD: 0.54–3.5 μg/kg LOQ: 1.8–12 μg/kg	[111]
Coffee	HS–GC/MS	LOD: 1.5–6.0 μg/kg LOQ: 5–20 μg/kg	[112]
Five types of nuts	HS–SPME–GC/MS	LOD: 0.09 μg/kg LOQ: 0.27 μg/kg	[113]
Dark chocolate	HS–SPME–GC/MS	LOD: 0.5 μg/kg LOQ: 1.5 μg/kg	[114]
Coffee	HS–SPME–GC/MS	LOD: 0.02 μg/kg LOQ: 0.06 μg/kg	[115]
Dried fruits and roasted nuts	HS–SPME–GC/MS	LOD: 0.012–0.425 μg/kg LOQ: 0.038–1.275 μg/kg	[116]

**Table 4:** List of analysis methods for AA in different food types.

Food Type	Analytical Techniques	Validation Parameters	References
Roasted almonds, raw ground pork, etc.	UPLC-MS/MS	LOD: 0.63 μg/L LOQ: 2.1 μg/L Recovery: 98.16–103.76%	[138]
Cookies, bread, potato crisps, milk, etc.	Novel colorimetric analysis and UV-Vis spectral sensing platform	LOD: 0.16 μM	[131]
Coffee, biscuit	Filtration- assisted optical detection	LOD: 14 μM	[145]
White bread	Carbon quantum dots	LOD: 2.6 μM	[146]
Potato chips	Dex-MMIPs/ HPLC-UV	LOD: 0.28 μM	[139]
Roasted nut samples	Three-way junction-engineered LC aptasensor	LOD: 0.106 amol/L Recovery: 96.84–99.61%	[137]
Potato chips samples	LC/MS-MS and FTIR		[140]
Biscuits, potato chips, etc.	UPLC-MS/MS	LOD: 0.63 μg/L LOQ: 2.1 μg/L Recovery: 98.16–103.76%	[138]
Roasted chicory sample	LC-MS/MS	LOD: 5 μg/kg LOQ: 15 μg/L Recovery: 95.86–103.06%	[134]

**Table 5:** List of analysis methods for TFAs in different food types.

Food Type	Analytical Techniques	Validation Parameters	References
Milk	NIR	R^2^_CV_ = 0.78 R^2^_V_ = 0.37	[172]
Milk	Miniaturized near-infrared spectrophotometer	RPD = 2.0–2.9	[173]
Muffalo milk	2-dimensional gas chromatography		[174]
Butters and margarines	Mid- and near-infrared spectrometers	SEP ≤ 2.62 RPD: 1.4–15.1 RER: 5.7–56.9	[175]
Rapeseed and soybean oil	Raman spectroscopy based on competitive adaptive reweighted sampling coupled with support vector regression		[176]
Edible oils	Raman spectroscopy and chemometric methods	R_cv_ = 0.9598, Rp = 0.9634, RMSEC = 0.351	[177]
Oleic and elaidic acids	Raman spectroscopy		[178]
Edible oils	Capillary electromigration methods		[179]
Edible oils	^1^H NMR spectroscopy		[180]
Meat	GC-FID		[181]
Commercial instant noodle	GC		[182]
Vegetable oils	GC-MS-MIQ		[183]

**Table 6:** List of analysis methods for AGEs in different food types.

Food Type	Analytical Techniques	Validation Parameters	References
Patties	HPLC-MS/MS	LOD: 0.19–3.58 μmol/kg LOQ: 0.56–10.74 mg/kg	[215]
Roasted chicken	ESI-LC-MS/MS	LOD: 0.30–19.02 ng/mL LOQ: 0.87–57.06 ng/mL Recovery: 71–110%	[223]
Industrial bread	LC-Electrospray Ionization-MS/MS	LOD: 0.02–0.17 mg/kg LOQ: 0.03–0.57 mg/kg	[224]
Butter cookies	HPLC-QqQ-MS/MS		[225]
Roast/Grilled meat	UPLC-MS/MS	LOD: 0.3–5.5 μg/L LOQ: 0.9–6.3 μg/L	[217]
Beef	High-throughput DART-MS/MS	LOD: 0.15 μg/g LOQ: 0.6 μg/g	[216]
Milk	UPLC-DAD	LOD: 0.50 g/g protein	[226]
Dairy products	HPLC-MS/MS	LOD: 0.1 μg/kg	[227]
Minced pork	HPLC-MS/MS	LOD: 4–5 g/L(CML); 12–15 g/L(CEL)	[228]
Mutton	LC-MS/MS	LOD: 3.6 ng/mL (CML); 1.9 ng/mL (CEL)	[229]
Bread	UPLC-MS/MS	LOD: 0.75 μg/kg (CML); 2.5 μg/kg (CEL)	[230]
Roasted beef patties	UPLC-MS/MS	LOD: 0.052 ng/g (CML); 0.098 ng/g (CEL)	[231]
Sterilized milk	UPLC-MS/MS	LOD: 0.05 mg/kg (CML)	[232]
Infant formula	ELISA	LOD: 550–600 ng/mL (CML)	[233]

**Table 7:** Major emerging technologies in the thermal processing of food and their impacts on the formation of contaminants in thermal processing.

Technology	Sample	Main Results	References
HPP	Table olives	HPP did not contribute to acrylamide formation.	[265]
HPP	Black garlic	HPP increased Maillard reaction.	[266]
US	Bovine serum albumin	US unfolded or enhanced aggregation behavior in protein samples, altering accessibility of lysine and arginine.	[267]
PEF	In vitro and in vivo assays	PEF reduced 4.8% the AGE content in diabetic mices.	[268]
Frying assisted with US and PEF	Potato chips	Use of US decreased the acrylamide content; the use of US coupled with PEF decreased even further the acrylamide content.	[269]
Air Frying	Chicken breast and grape seed extracts	Air frying combined with grape seed extracts inhibited the formation of AGEs (CML and CEL), its precursors (GO, MGO) and increased oxidative stability. Air frying, even without the addition of grape seed extracts, promoted less CML, CEL, GO and MGO formation.	[270]
Vacuum Frying	French fries and frying oil	Reduced formation of acrylamide content.	[271]

## Data Availability

No new data were created or analyzed in this study. Data sharing is not applicable to this article.
